# Prevalence, Symbiosis with *Rickettsia*, and Transmission of *Tomato yellow leaf curl virus* of Invasive *Bemisia tabaci* MED Q2 in Japan

**DOI:** 10.1264/jsme2.ME24095

**Published:** 2025-05-01

**Authors:** Akiko Fujiwara, Hiroki Hagiwara, Maiko Tsuchimoto, Tsutomu Tsuchida

**Affiliations:** 1 Center for Food Science and Wellness, Gunma University, 4–2 Aramaki, Maebashi, Gunma 371–8510 Japan; 2 Graduate School of Science and Engineering for Education, University of Toyama, 3190 Gofuku, Toyama, Toyama, 930–8555, Japan; 3 Faculty of Science, Academic Assembly, University of Toyama, 3190 Gofuku, Toyama, Toyama, 930–8555, Japan

**Keywords:** *Portiera*, *Rickettsia*, endosymbiotic bacteria, invasive pest insect, virus transmission

## Abstract

The whitefly, *Bemisia tabaci*, is a notorious insect pest that transmits plant pathogenic viruses to a wide range of economically important crops. An invasive genetic group of *B. tabaci*, Mediterranean Q2 (MED Q2), has recently spread to Europe, USA, and Asia. In the present study, we investigated the prevalence of MED Q2 in Japanese agricultural sites and found that its distribution has expanded since it was initially detected in 2013. A polymerase chain reaction ana­lysis revealed that all MED Q2 individuals were infected with *Rickettsia*. *Rickettsia* titers increased during nymphal development, presumably in response to the nutritional needs of the host. A fluorescence *in situ* hybridization ana­lysis revealed that *Rickettsia* was densely located near *Portiera*-containing bacteriocytes at all growth stages. Therefore, *Rickettsia* may play an important role, such as supplying nutrients to the host, in cooperation with *Portiera*. Transfer experiments indicated that MED Q2 was as effective a vector for *Tomato yellow leaf curl virus* as MED Q1 and, thus, is a high-risk agricultural pest. These results provide important insights into the biology and ecology of invasive MED Q2 to effectively control its spread and minimize its impact on crops.

Globalization and international trade facilitate the long-distance movement of non-native organisms beyond their natural geographic range (Chapman *et al.*, 2017; Epanchin-Niell *et al.*, 2021). The invasion of alien species has increased in recent decades (Seebens *et al.*, 2017), with detrimental effects on human health, ecosystems, and economic activities, such as agriculture (Simberloff *et al.*, 2013; Pyšek and Richardson, 2010).

The sweet potato whitefly *Bemisia tabaci* (Hemiptera: Aleyrodidae) is a phloem sap-feeding pest of agricultural crops (Stansly and Naranjo, 2010). It comprises genetically distinct groups that exhibit different biological characteristics, such as habitat and susceptibility to insecticides, but are morphologically indistinguishable (Costa and Brown, 1991; Bedford *et al.*, 1994). Based on mitochondrial cytochrome oxidase subunit I (*mtCOI*) gene sequences, it may be classified into 44 groups (Kanakala and Ghanim, 2019). Although many genotypes are limited to specific regions, Mediterranean (MED) Q (Moya *et al.*, 2001; Boykin *et al.*, 2007) and Middle East-Asia Minor 1 (MEAM1) (Frohlich *et al.*, 1999; De Barro *et al.*, 2000) have spread globally via international transport from their original habitats in the Mediterranean region and the Middle East and Asia Minor, respectively (De Barro and Ahmed, 2011). A common characteristic of these invasive genetic groups is their high insecticide resistance. This may explain why they have expanded their range in agricultural fields and greenhouses, where higher amounts of insecticides are used, and have replaced existing indigenous species (Brown *et al.*, 1995; Horowitz *et al.*, 2005; Nauen and Denholm, 2005). These genetic groups may cause substantial damage to a wide range of economically important crops by transmitting more than 100 pathogenic plant viruses (Jones, 2003; Hogenhout *et al.*, 2008). Due to these characteristics, the whitefly, represented by these genotypes, is regarded as one of the most seriously invasive species worldwide according to the global invasive species database (Lowe *et al.*, 2000).

MED Q has been classified into three subgroups, Q1, Q2, and Q3, based on mole­cular phylogenetic ana­lyses of the *mtCOI* gene (Gueguen *et al.*, 2010). MED Q2 originated in the Eastern Mediterranean and has expanded its distribution in Europe and the United States (Tsagkarakou *et al.*, 2007; Chu *et al.*, 2008; Ahmed *et al.*, 2009; Mouton *et al.*, 2012; Parrella *et al.*, 2014; Karut *et al.*, 2017). In recent years, the distribution of MED Q2 has expanded to Asia. MED Q2 was initially discovered in Asia in 2013 at a site in the Kanto region of Japan (Fujiwara *et al.*, 2015). In 2015, MED Q2 was identified at two additional sites in the same district of Japan (Kurata *et al.*, 2016). This included one site in which MED Q2 was not detected in the 2013 survey. In 2018, MED Q2 was initially identified at two sites in northwestern and southeastern South Korea (Guo *et al.*, 2021). These findings suggest that the range of MED Q2 continues to expand.

Similar to other phloem-sap feeding insects, *B. tabaci* harbors endosymbiotic bacteria that are stably transmitted to offspring via the ovaries (Buchner, 1965; Luan *et al.*, 2018). All genetic groups of *B. tabaci* contain the primary endosymbiont, *Portiera*. *Portiera* is regarded as an ancient infection with an insect ancestor (Baumann *et al.*, 2004; Thao and Baumann, 2004). In addition to *Portiera*, *B. tabaci* often harbors different endosymbiotic bacteria. Eight different bacteria, *Hamiltonella*, *Cardinium*, *Rickettsia*, *Wolbachia*, *Arsenophonus*, *Frischea*, *Hemipteriphilus*, and *Candidatus* Rickettsia_Torix_Bemisia_tabaci, have been reported to date (Zchori-Fein and Brown, 2002; Nirgianaki *et al.*, 2003; Weeks *et al.*, 2003; Baumann, 2005; Everett *et al.*, 2005; Gottlieb *et al.*, 2006; Bing *et al.*, 2013b; Wang *et al.*, 2020). These bacteria are considered to have been acquired relatively recently and are collectively referred to as secondary symbionts (S-symbionts).

The MED Q1 and MEAM1 genotypes harbor *Portiera* and *Hamiltonella* exclusively within bacteriocytes, which are specialized cells for endosymbiosis (Gottlieb *et al.*, 2008; Skaljac *et al.*, 2010; Li *et al.*, 2022). In these genotypes, *Portiera* and *Hamiltonella* are regarded as co-obligate symbionts based on virtually universal infection in their original populations (Gueguen *et al.*, 2010; Tsagkarakou *et al.*, 2012; Gnankiné *et al.*, 2013; Bing *et al.*, 2013a; Fujiwara *et al.*, 2015; Zchori-Fein *et al.*, 2014; Marubayashi *et al.*, 2014; Karut *et al.*, 2017) and the decrease in host fitness caused by symbiont elimination (Su *et al.*, 2013, 2014). However, studies conducted on Italian, Israeli, Turkish, and Japanese populations revealed the absence of *Hamiltonella* in MED Q2 (Chiel *et al.*, 2007; Gueguen *et al.*, 2010; Karut and Tok, 2014; Parrella *et al.*, 2014; Karut *et al.*, 2017; Fujiwara *et al.*, 2015; Kurata *et al.*, 2016). *Rickettsia* is nearly fixed in all MED Q2 populations, suggesting its important role in replacing *Hamiltonella* in MED Q2. Previous studies reported various effects of *Rickettsia* on naturally infected MEAM1, such as increased susceptibility to insecticides (Kontsedalov *et al.*, 2008), a greater capacity to transmit *Tomato yellow leaf curl virus* (TYLCV) (Kliot *et al.*, 2014), more fitness benefits, and a higher female-biased sex ratio (Himler *et al.*, 2011). In contrast, limited information is available on the effects of *Rickettsia* on MED Q2.

In the present study, we characterized MED Q2 and its secondary symbiont, *Rickettsia*. We conducted a detailed survey on the distribution of MED Q2 and its infection status with symbiotic bacteria in the Japanese population. We then analyzed the spatiotemporal dynamics of *Rickettsia* in MED Q2 using quantitative PCR (qPCR) and *in situ* hybridization. We also exami­ned the retention of TYLCV in the body and the efficiency of its transmission to tomato plants in MED Q2.

## Materials and Methods

### Insects and plants

Whiteflies were collected from fields in the Kanto region of Japan between 2016 and 2021 (Table S1 and Fig. S1). All samples were immediately stored in 100% acetone for later ana­lyses (Fukatsu, 1999). Laboratory strains of *B. tabaci* MED Q1 with *Portiera*, *Hamiltonella*, and *Cardinium* (Fujiwara *et al.*, 2023) and MED Q2 with *Portiera* and *Rickettsia* (strain Maebashi, Locality no. 14 in Table S1) were used in the present study. The genotypes of these strains and their symbionts were confirmed by sequencing, as previously described (Fujiwara *et al.*, 2015). These strains were maintained on cabbage (*Brassica oleracea*) leaves at 25±1°C and 40–60% relative humidity in a long-day regimen (16‍ ‍h light, 8‍ ‍h dark), and exhibited a sex ratio of approximately 1:1. Two tomato plants (*Solanum lycopersicum*), ‘Momotaro’ (Takii) and ‘Micro-Tom’ (provided by the National Bio-Resource Project [NBRP] tomato program at the University of Tsukuba, Japan), were cultivated under the same temperature and humidity conditions as cabbage and used in infection experiments with TYLCV. TYLCV-Israel and Mild strains (TYLCV-IL and TYLCV-Mld) were infected and maintained in tomato plants (cv. House-Momotaro) (Takii). These cabbage and tomato plant species are not listed as endangered species or species at risk of extinction, according to the IUCN Policy Statement and the Convention on International Trade in Endangered Species of Wild Fauna and Flora.

### Identification of genetic groups and symbionts of *B. tabaci*

Experiments and classifications using multiplex PCR were performed as previously described (Kurata *et al.*, 2016). Following the extraction of the total DNA of *B. tabaci* and its symbionts from the whole body of each insect, it was amplified using three sets of primer mixes (mix for genotyping, mix 1 for symbionts, and mix 2 for symbionts), as listed in Table S2. *mtCOI* gene sequences were elucidated for 11 representative samples as previously described (Fujiwara *et al.*, 2015) with minor modifications. Partial sequences were amplified using PCR with KOD FX Neo (TOYOBO) using specific primer sets (Table S2). PCR products were gel-purified and sequenced directly. Sequences were assembled using Geneious Prime ver. 2020.0.5 software (Dotmatics). Sequence similarities in the detected genotypes were analyzed using BLAST (Altschul *et al.*, 1990).

### qPCR

Eleven individuals of the MED Q2 laboratory strain were collected at each developmental stage from egg to adult. Eggs and first to fourth instar nymphs were collected without distinguishing between males and females because the sex of whiteflies is indistinguishable at immature stages. At the adult stage, whiteflies of both sexes were collected separately. Samples were preserved in 100% acetone (Fukatsu, 1999) until DNA extraction. DNA was extracted from individual samples using a NucleoSpin Tissue XS Kit (Takara Bio). DNA was extracted from the eggs of 10 individuals. *Portiera* and *Rickettsia* were quantified in terms of 16S rRNA or *gltA* gene copies using the CFX Connect Real-Time PCR Detection System (Bio-Rad Laboratories) with KOD SYBR qPCR Mix (Toyobo) and specific primer sets (Table S2). qPCR conditions were as follows: at 98°C for 2‍ ‍min, followed by 40 cycles at 98°C for 10‍ ‍s, each annealing temperature for 10‍ ‍s, and a final extension at 68°C for 30 s. qPCR with a dissociation curve ana­lysis was conducted using a standard curve method, as previously described (Tsuchida *et al.*, 2014). TYLCV was quantified using the same system targeting *v1*, with specific primer sets (Table S2). qPCR conditions were as follows: at 98°C for 2‍ ‍min, followed by 40 cycles at 98°C for 10‍ ‍s and 65°C for 10‍ ‍s, and a final extension at 68°C for 30 s.

### Fluorescence *in situ* hybridization (FISH)

Whole-body or dissected insect tissue specimens were fixed in Carnoy’s solution (EtOH: chloroform: glacial acetic acid, 6:3:1), bleached in 6% hydrogen peroxide in EtOH, and subjected to whole-mount FISH, as previously described (Koga *et al.*, 2009). Fluorochrome-labeled oligonucleotide probes are listed in Table S2. Host cell nuclei were counterstained with 4,6-diamino-2-phenylindole (DAPI). Observations were performed using a laser scanning confocal microscope (LSM880; Carl Zeiss) and analysed using LSM ZEN2 software (Carl Zeiss). The specificity of *in situ* hybridization was confirmed by the following control experiments: a no-probe control and an RNase digestion control, as previously described (Tsuchida *et al.*, 2014).

### Comparison of TYLCV retention in MED Q1 and Q2

Experiments were performed as previously described (Li *et al.*, 2010) with modifications. Approximately 30 adult whiteflies (3–4‍ ‍d after eclosion) of MED Q1 or MED Q2 were released on each of the two TYLCV (IL or Mld)-infected tomato plants in a plastic container with an insect-proof mesh. Insects were allowed to feed on the plants for 48‍ ‍h to acquire TYLCV. Total DNA was extracted from each individual of *B. tabaci* using a simple extraction method as previously described (Kurata *et al.*, 2016). The amount of TYLCV in the samples was measured using qPCR, as described above.

### Transmission of TYLCV

Approximately 200 adult whiteflies (MED Q1 or MED Q2), 3–4‍ ‍d after eclosion, were released on each of the two TYLCV (IL or Mld)-infected tomato plants in a plastic container with an insect-proof mesh. Insects were allowed to feed on the plants for 48‍ ‍h in order to acquire the TYLCV strain. Ten adults were collected and transferred to a healthy tomato plant (cv. Momotaro, or Micro-Tom) with three true leaves. Insects were allowed to inoculate tomato plants with TYLCV for 48 or 72 h. As described in a previous study (Mehta *et al.*, 1994), we referred to this period as the inoculation access period (IAP) (Table 1). Experiments were repeated ten times. Inoculated plants were kept at 25±1°C and 40–60% relative humidity in a long-day regimen (16‍ ‍h light, 8‍ ‍h dark). After 30‍ ‍d, we checked the plants for symptoms of the disease (*i.e.*, yellowing, leaf curling, and dwarfing). We then collected the youngest leaves from each plant for DNA extraction, following a previously described method (Thomson and Henry, 1995). The presence and type of TYLCV were confirmed using multiplex PCR with the specific primers listed in Table S2. PCR conditions were as follows: at 95°C for 5‍ ‍min, followed by 35 cycles at 98°C for 10‍ ‍s and 55°C for 30‍ ‍s, and a final extension at 68°C for 60 s.

### Statistical ana­lysis

The Wilcoxon rank-sum test after the Bonferroni correction was used to evaluate differences in the bacterial titers of *Portiera* and *Rickettsia* between the host sexes. A two-way ana­lysis of variance (ANOVA) using generalized linear modeling (GLM) with a Poisson error structure was adopted to examine the effects of the host sex (female or male), the genetic group of the host (MED Q1 or MED Q2), and their interactions on TYLCV titers. Fisher’s exact test was used to investigate differences in the transmission rates of TYLCV between MED Q1 and MED Q2. This ana­lysis was conducted for each TYLCV-IL and TYLCV-Mld strain. All statistical ana­lyses were performed using R software v. 4.2.1. (Ihaka and Gentleman, 1996).

### Accession numbers

All sequences elucidated in the present study were deposited in the DDBJ/NCBI/GenBank database under the following accession numbers: LC735029–LC735039 for *mtCOI* and LC795726 and LC795727 for the 16S rRNA genes of symbionts of the laboratory MED Q2 strain.

## Results

### Prevalence of MED Q2

**Fig. 1.**
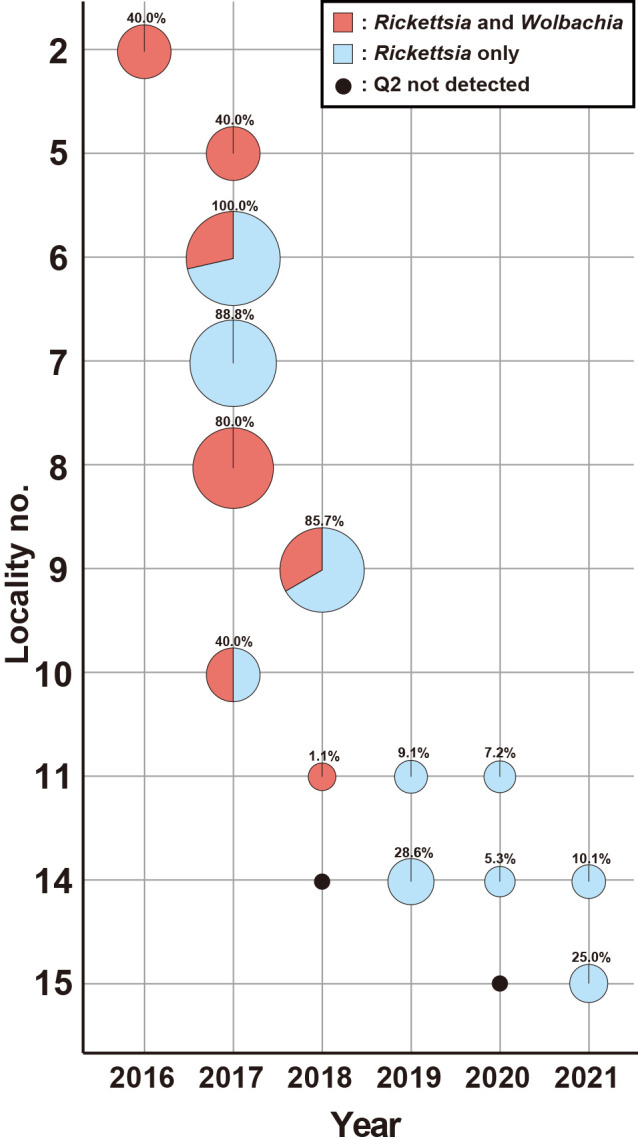
Changes in the prevalence of MED Q2 and its infection status with secondary symbionts (S-symbionts) in the Kanto region of Japan. Locality numbers correspond to those in Fig. S1 and Table S1. Data indicate the total number of MED Q2 individuals collected from various crops listed in Table S1. The percentage of MED Q2 (number of individuals/total number of whiteflies exami­ned) is indicated by the size of the pie chart. The percentage value is at the top of the pie chart. The black dot indicates that the survey was conducted and MED Q2 was not detected. The infection status of S-symbionts is shown in different colors: light red, infection with *Rickettsia* and *Wolbachia*; sky blue, infection with *Rickettsia* only.

We collected* B. tabaci* samples from 15 sites in the Kanto region of Japan (Fig. S1) between 2016 and 2021. Multiplex PCR detected three genetic groups: MED Q1, MED Q2, and JpL (Table S1). MED Q1 was detected in 14 sites. JpL was only detected in two sites (localities 14 and 15). MED Q2 was detected in 10 of the 15 sites. The identification of MED Q2 was confirmed using partial *mtCOI* sequencing. MED Q2 was detected at high frequencies at five of the 10 sites, with regional differences ranging between 100% and 1.1% (Table S1). Multi-year surveys were conducted at three sites in Gunma Prefecture: Isesaki (site 11), Maebashi (site 14), and Yoshioka (site 15). MED Q2 was not detected in Maebashi or Yoshioka in the first year of the survey, but was identified in the same sites in the following year (Table S1 and Fig. 1). MED Q2 was found at moderate rates in Isesaki and Maebashi over three years; however, the number of MED Q2 individuals varied among years and sites.

### Endosymbiotic microbiota of MED Q2

Multiplex PCR detected *Rickettsia* in all individuals in the MED Q2 population, in addition to *Portiera* (Fig. 1). Some MED Q2 also harbored *Wolbachia*. The infection rate‍ ‍of *Wolbachia* varied between regions or collection dates; *Wolbacia* was never detected at some sites (no. 14 and 15, Fig. 1). The S-symbionts, *Hamiltonella*, *Cardinium*, *Arsenophonus*, and *Hemipteriphilus*, were not detected in any of the individuals exami­ned.

### Population dynamics of *Portiera* and *Rickettsia* in host developmental stages

**Fig. 2.**
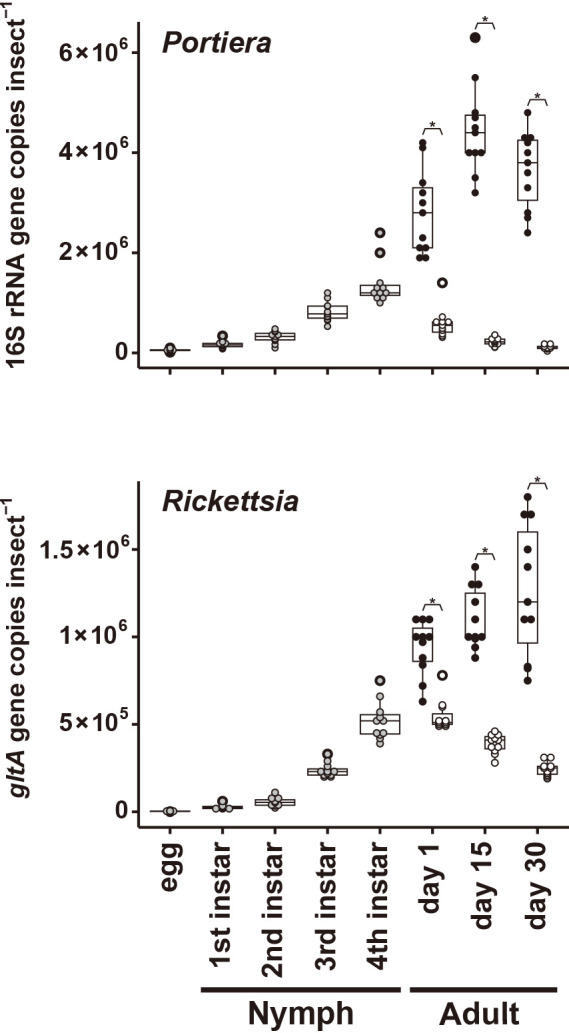
Population dynamics of symbionts in *Bemisia tabaci* MED Q2. Bacterial titers of *Portiera* and *Rickettsia* were measured using a quantitative polymerase chain reaction in terms of 16S rRNA or *gltA* gene copies insect^–1^. Each dot represents an individual; grey circles, eggs and nymphs; filled circles, adult females; open circles, adult males; *n*=10 for eggs, *n*=11 for others. The symbiont titer in an egg was calculated by averaging the acquired values of 10 individuals. Asterisks indicate a significant difference (*P*<0.001, the Wilcoxon rank-sum test after the Bonferroni correction).

*Portiera* titers continued to increase throughout the nymphal stages, peaked in fourth instar nymphs in males and on day 15 in adult females (Fig. 2), and then began to decrease. *Rickettsia* showed similar population dynamics to *Portiera*, with titers increasing throughout the nymphal stage in both females and males. The bacterial population was markedly lower in males than in females at all adult stages (Fig. 2). In males, *Rickettsia* populations decreased rapidly from 1‍ ‍d after adult eclosion, but remained high in females 30‍ ‍d after eclosion.

### *In vivo* localization of symbionts in MED Q2

**Fig. 3.**
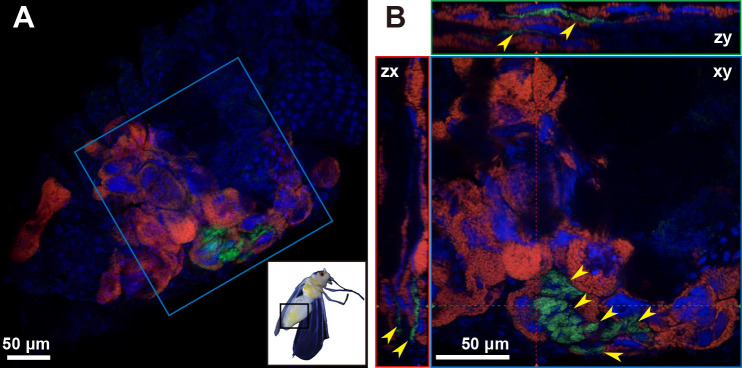
*In vivo* localization of *Portiera* (red) and *Rickettsia* (green) in *Bemisia tabaci* MED Q2. (A) The abdomen of a female 15‍ ‍d after eclosion. A whole view of *B. tabaci* is shown in the lower right. The area enclosed by the black square was observed. (B) Enlarged image of the area indicated by the blue square in (A). Host nuclear DNA is visualized in blue. Yellow arrowhead, *Rickettsia*, densely localized in close proximity to bacteriocytes. In (B), orthogonal views of Z-stack images are shown; red and green dashed lines indicate corresponding points in the orthogonal planes.

In adult females, *Portiera* was only detected in bacteriocytes (Fig. 3A and B). In contrast to *Portiera*, *Rickettsia* was not detected in bacteriocytes. This distribution pattern was confirmed by observing bacteriocytes dissected from adult females 1‍ ‍d after eclosion (Fig. S2). *Rickettsia* was densely localized in close proximity to bacteriocytes, and was rarely observed in other tissues (Fig. 3A and B). Similar localization patterns were observed in first-instar, fourth-instar, and adult males 1‍ ‍d after eclosion (Fig. S3).

**Fig. 4.**
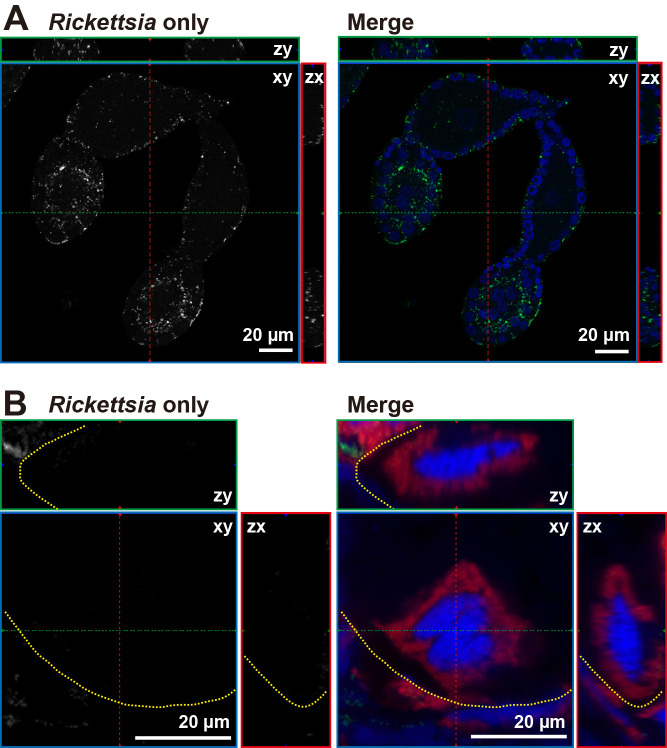
Localization of *Rickettsia* in the ovary of MED Q2. (A) Ovarioles dissected from an adult female 5‍ ‍d after eclosion. (B) A bacteriocyte just after entering the egg in an adult female 15‍ ‍d after eclosion. Only the *Rickettsia* (white) signal is shown in the left images. The right images show the nuclear DNA (blue) and *Portiera* (red) signals overlaid on the *Rickettsia* (green) signal. Yellow dashed lines indicate outlines of the egg. An orthogonal view of Z-stack images is shown. Red and green dashed lines indicate corresponding points in the orthogonal planes.

Ovaries collected from adult females 5‍ ‍d after eclosion contained eggs at the pre-vitellogenesis stage (Fig. 4A) before the transfer of bacteriocytes. A FISH ana­lysis of ovaries showed that *Rickettsia* was present in the eggs at this stage (Fig. 4A). On day 15 after adult eclosion, bacteriocytes containing *Portiera* were detected in the ovaries (Fig. 4B). However, *Rickettsia* was not detected within or on bacteriocytes. These results indicate that *Rickettsia* of MED Q2 was not present inside bacteriocytes and was not transmitted to the next generation via bacteriocytes, in contrast to *Portiera*.

### Retention and transmission of TYLCV in MED Q2

**Fig. 5.**
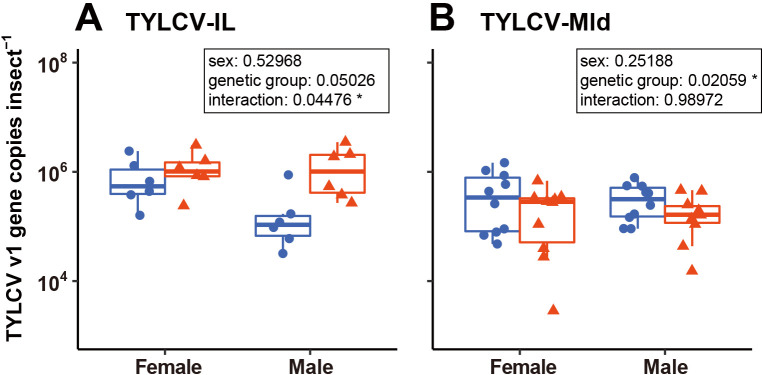
Effects of host sex and genotype on the retention of TYLCV-IL (A) and TYLCV-Mld (B). Box plots represent the distribution of TYLCV titers insect^–1^. Each dot represents an individual value. Blue circles, MED Q1; red triangles, MED Q2; Six and 10 individuals per group were used for the TYLCV-IL and TYLCV-Mld experiments, respectively. The results of a two-way ANOVA using a generalized linear model with a Poisson error structure are shown in the box (*P<0.05).

Fig. 5 shows TYLCV titers in *B. tabaci* after 48‍ ‍h of feeding on TYLCV-infected plants. A two-way ANOVA using GLM showed that TYLCV-IL titers were slightly higher in MED Q2 than in MED Q1. An interaction was observed between sex and genetic groups. In contrast, TYLCV-Mld titers were significantly lower in MED Q2 than in MED Q1 (Fig. 5). Regarding TYLCV-Mld, no interaction was observed between sex and genetic groups.

The PCR ana­lysis conducted in transfer experiments showed that TYLCV-IL was transferred to Momotaro or Micro-Tom by MED Q1 and MED Q2 at similar levels under 48-h IAP conditions (Table 1). When IAP was extended to 72‍ ‍h in Momotaro, the transmission rate of TYLCV-IL increased in both MED Q2 and MED Q1. When TYLCV-Mld was transferred to Micro-Tom under 48-h IAP conditions, MED Q2 was slightly less efficient than MED Q1. When transferred to Momotaro for 72 h, MED Q2 efficiency was 100% and was slightly more efficient than MED Q1. Disease symptom results were similar to those of TYLCV PCR detection. No significant differences were found in transmission rates between the MED Q1 and MED Q2 groups (Table 1).

## Discussion

MED Q2 was initially detected in 2013 at a site in the Kanto region of Japan (Fujiwara *et al.*, 2015). In 2015, MED Q2 was detected at two additional sites in the same area (Kurata *et al.*, 2016). In the present study, we detected MED Q2 at ten sites in the same district (Table S1). In a previous study, we also investigated two sites, Isesaki (locality no. 11) and Maebashi (locality no. 14), but did not detect MED Q2 (0/12 and 0/6 individuals, respectively) (Fujiwara *et al.*, 2015). These findings suggest that MED Q2 has expanded its distribution area in the Kanto region since 2013; however, MED Q1 is still the dominant strain in Japan. In the present study, MED Q2 was detected at Sites 11 and 14 over multiple years (Table S1 and Fig. 1), suggesting that the MED Q2 population was established here and in the wider area of the Kanto region. However, it is important to note that in this survey, the number of individuals sampled was small at some sites, which may have led to an underestimation of the actual population size of MED Q2. Therefore, additional surveys with larger sample sizes are needed to further clarify the distribution and establishment of MED Q2.

MED Q2 appeared to have expanded its distribution to many countries. In South Korea, MED Q2 was initially identified in 2018 (Guo *et al.*, 2021). In southern Italy, the distribution of MED Q2 has expanded since it was first discovered, and the dominant type changed from MED Q1 to MED Q2 in only a few years (2010–2013) (Parrella *et al.*, 2014). In the 2017–2019 surveys conducted in central and southern Italy and Sicily, MED Q2 accounted for 87.6% of all samples collected from greenhouse crops (Bertin *et al.*, 2021). The high temperature tolerance, insecticide resistance, and female-biased sex ratio of MED Q2 are considered to have contributed to its spread in Italy (Parrella *et al.*, 2014). Since a female-biased sex ratio was not observed in the Japanese MED Q2 strain (see the Materials and Methods section), global warming and insecticide use may be responsible for the expanding distribution of MED Q2 in Japan. To address this possibility, future studies need to investigate the distribution of MED Q2 in regions with varying annual temperatures and insecticide usage across wider areas of Japan. Additionally, to prevent the spread of MED Q2, effective insecticides and other methods must be identified.

The infection status of MED Q2 was previously reported in Israel (Chiel *et al.*, 2007; Gueguen *et al.*, 2010), Turkey (Karut and Tok, 2014; Karut *et al.*, 2017), and Italy (Parrella *et al.*, 2014). MED Q2 in these countries commonly had a high infection rate with *Arsenophonus* and *Rickettsia*, but a low infection rate with *Wolbachia*. The infection status of the Japanese MED Q2 group markedly differed from those of the other groups. In addition to the primary symbiont, *Portiera*, two types of S-symbionts, *Rickettsia* and *Wolbachia*, were detected in Japanese MED Q2 (Fig. 1). The prevalence of *Rickettsia* infection was 100% throughout all survey periods; *Wolbachia* was only detected in some samples. *Arsenophonus*, which is highly prevalent in MED Q2 in other countries, has never been detected in the Japanese MED Q2 population. Possible reasons for this difference are as follows: (1) only MED Q2, which is uninfected with *Arsenophonus*, has entered Japan, and (2) the population of *Arsenophonus*-uninfected MED Q2 has increased due to its better adaptability to Japanese field conditions. However, these hypotheses are not mutually exclusive. A more detailed ana­lysis is needed in the future to clarify this issue.

*Rickettsia* was nearly fixed in MED Q2 populations in all regions investigated in this study. *Rickettsia* infection may increase the percentage of infected individuals by manipulation of the sex ratio, as demonstrated in MEAM1 (Himler *et al.*, 2011). Another possibility is that *Rickettsia* promoted survival and reproduction in MED Q2 through nutritional supply, thereby increasing the number of infected individuals in the population. The second possibility is based on its population dynamics in host insects; *Rickettsia* titers increased during nymphal development and then rapidly decreased in males from 1‍ ‍d after eclosion (Fig. 2). These bacteria population dynamics have been observed in many obligate symbionts of various insect hosts, including *Buchnera* in the pea aphid (*Acyrthosiphon pisum*), *Sodalis* in cereal weevils (*Sitophilus oryzae*), and *Portiera* and *Hamiltonella* in *B. tabaci* MEAM1 (Koga *et al.*, 2003; Sakurai *et al.*, 2005; Simonet *et al.*, 2018, Fujiwara *et al.*, 2023). This phenomenon has been interpreted as the regulation of nutrient-compensating symbionts to meet the dietary needs of host insects. The *Rickettsia* titer in females remained at higher levels for a longer duration than that in males (Fig. 2). This suggests that symbiotic populations are regulated differently depending on the sex of the host. It is conceivable that higher *Rickettsia* levels in females evolved to ensure vertical transmission, as proposed for the obligate symbiont *Hamiltonella* in the MEAM1 of *B. tabaci* (Fujiwara *et al.*, 2023).

Previous genomic studies on *B. tabaci* MEAM1 and MED Q1 showed that *Portiera* alone was unable to synthesize essential amino acids (*e.g.*, lysine) or B vitamins that are deficient in the host diet (Santos-Garcia *et al.*, 2015; Chen *et al.*, 2016; Xie *et al.*, 2018); the enzymes responsible for producing essential amino acids are found across *Portiera*, a coexisting symbiont (*Hamiltonella* or *Arsenophonus*), and *B. tabaci*. Therefore, metabolic intermediates must be transported between symbionts and the host multiple times for production. These symbionts coexist within the same bacteriocyte (Gottlieb *et al.*, 2008), which appears to be adaptive for the efficient production of essential nutrients via intertwined metabolic pathways (Fujiwara *et al.*, 2023). Incomplete metabolic pathways were found in *Portiera* for both MEAM1 and MED Q1 (Santos-Garcia *et al.*, 2012; Sloan and Moran, 2012), suggesting that the loss of these enzymes in *Portiera* occurred before the divergence of these lineages. Therefore, *Portiera* may have incomplete metabolic pathways in the derived lineage MED Q2. In the present study, *Rickettsia* was not detected in the bacteriocytes of MED Q2 (Fig. 3, 4, S2, and S3), which is in contrast to the previously reported “confined type” in which *Rickettsia* localized exclusively within bacteriocytes, as observed in MEAM1 and MED (Gottlieb *et al.*, 2008). Instead, *Rickettsia* aggregated in regions proximal to *Portiera*-containing bacteriocytes at all growth stages (Fig. 3A, B, and S3). The physical proximity of *Rickettsia* and *Portiera* in MED Q2 suggests a close interaction via nutrient metabolism. To identify their metabolic pathways and test this possibility, genomic ana­lyses of *Rickettsia* and *Portiera* in MED Q2 are required.

In MED Q1 and MEAM1, the co-obligate symbionts *Portiera* and *Hamiltonella* are passed on to the next generation by transferring a whole bacteriocyte bearing the symbionts to the developing egg (Luan *et al.*, 2018). *Rickettsia* in MED Q2 were detected in pre-vitellogenic oocytes (stage 1 egg as defined in Shan *et al.*, 2021), which is the stage before the bacteriocyte enters the egg. A previous study reported that *Rickettsia* in MEAM1 attached to a bacteriocyte and was transferred with the bacteriocyte to the developing egg (Shan *et al.*, 2021). However, in MED Q2, we did not observe *Rickettsia* on bacteriocytes in eggs (Fig. 4). This result suggests that there is a mechanism in MED Q2 for the transmission of *Rickettsia* into eggs without the involvement of bacteriocytes. In the future, the invasion process of *Rickettsia* into early developing eggs needs to be clarified in more detail using confocal and electron microscopy.

The most severe damage to crops caused by *B. tabaci* is due to the transmission of TYLCV. Therefore, the ability to transmit TYLCV is a critical factor in assessing the risk of whiteflies becoming an agricultural pest. According to previous studies, the ability of Israeli MED Q2 to transmit TYLCV is low because it lacks infection with *Hamiltonella*, the GroEL of which facilitates TYLCV transmission (Morin *et al.*, 1999, 2000; Gottlieb *et al.*, 2010; Ghanim, 2014). This study showed that Japanese MED Q2, which is free of *Hamiltonella* infection, is an effective vector for both TYLCV-IL and TYLCV-Mld, similar to *Hamiltonella*-infected MED Q1. MED Q2 showed the higher retention of TYLCV-IL (Fig. 5) as well as higher transmission rates to plants (Table 1) than MED Q1. TYLCV-Mld retention was significantly lower in MED Q2 than in MED Q1 (Fig. 5). However, transmission rates in MED Q2 were as high as those in MED Q1 (Table 1). These results indicate that Japanese MED Q2 is a high-risk agricultural pest, similar to‍ ‍MED Q1, and possesses efficient TYLCV transmis­sion‍ ‍machinery independent of *Hamiltonella*. *Rickettsia* may play a critical role in transmission. In a previous study on MEAM1, *Rickettsia* was suggested to accelerate the uptake of TYLCV into the hemolymph, which may enhance TYLCV transmission to tomato plants (Kliot *et al.*, 2014). Some proteins, such as GroEL, produced by *Rickettsia*, may be involved in the transmission efficiency of TYLCV in MED Q2, similar to *Hamiltonella* GroEL in MED Q1 (Gottlieb *et al.*, 2010). To investigate the potential role of *Rickettsia* in the high efficiency of TYLCV transmission in Japanese MED Q2, future experiments will require the elimination of *Rickettsia* through antibiotic treatment and an ana­lysis of its GroEL interaction with TYLCV. A more detailed understanding of the mechanisms underlying TYLCV transmission may lead to the development of more effective control technologies.

## Citation

Fujiwara, A., Hagiwara, H., Tsuchimoto, M., and Tsuchida, T. (2025) Prevalence, Symbiosis with *Rickettsia*, and Transmission of *Tomato yellow leaf curl virus* of Invasive *Bemisia tabaci* MED Q2 in Japan. *Microbes Environ ***40**: ME24095.

https://doi.org/10.1264/jsme2.ME24095

## Supplementary Material

Supplementary Material

## Figures and Tables

**Table 1.** Transmission rates of TYLCV in MED Q1 and Q2.

TYLCV strain^a^	IAP^b^	Variety of tomato (recipient)	Transmission rate (%)					
			PCR detection		*P* ^c^	Disease symptom		*P* ^c^
			MED Q1	MED Q2		MED Q1	MED Q2	
IL	48 h	Momotaro	50	50	1	50	50	1
IL	48 h	Micro-Tom	60	70	1	60	70	1
IL	72 h	Momotaro	70	90	0.582	60	80	0.629
Mld	48 h	Micro-Tom	30	10	0.582	30	10	0.582
Mld	72 h	Momotaro	70	100	0.211	70	100	0.211

^a^ IL, TYLCV-Israel strain; Mld, TYLCV-Mild strain ^b^ Inoculation access period ^c^ The results of Fisher’s exact test are shown.
