# Supplementary material for: Prevalence, Symbiosis with Rickettsia, and Transmission of Tomato yellow leaf curl virus of Invasive Bemisia tabaci MED Q2 in Japan
Source: Microbes Environ. 2025 May 1;40(2):ME24095. doi: 10.1264/jsme2.ME24095 (PMC12213066; doi:10.1264/jsme2.ME24095)
Supplement: Supplementary file 1 — Supplementary Material [file 40_24095_s1.pdf]

Supplementary Fig. S1

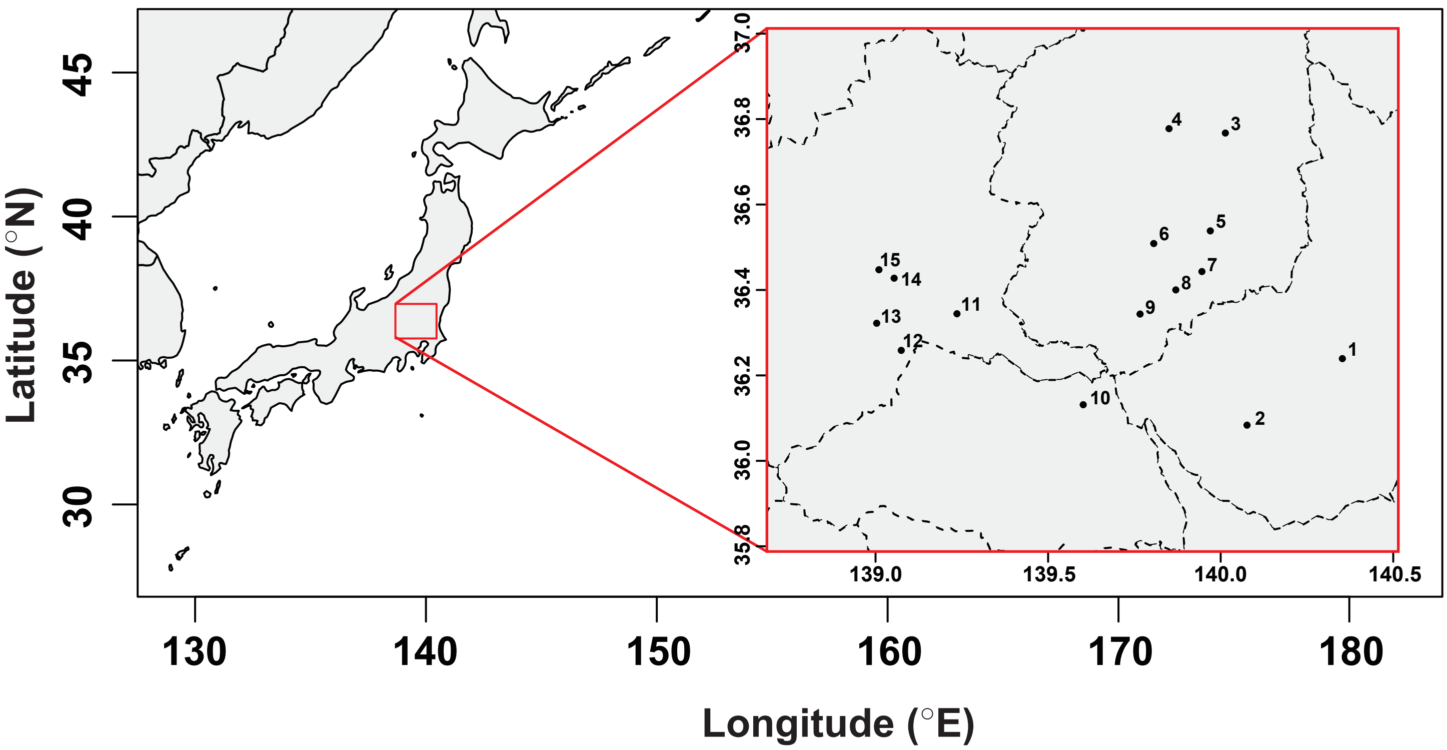

Fig. S1. Geographic distribution of *B. tabaci* sampling sites in the Kanto district of Japan.

Supplementary Fig. S2

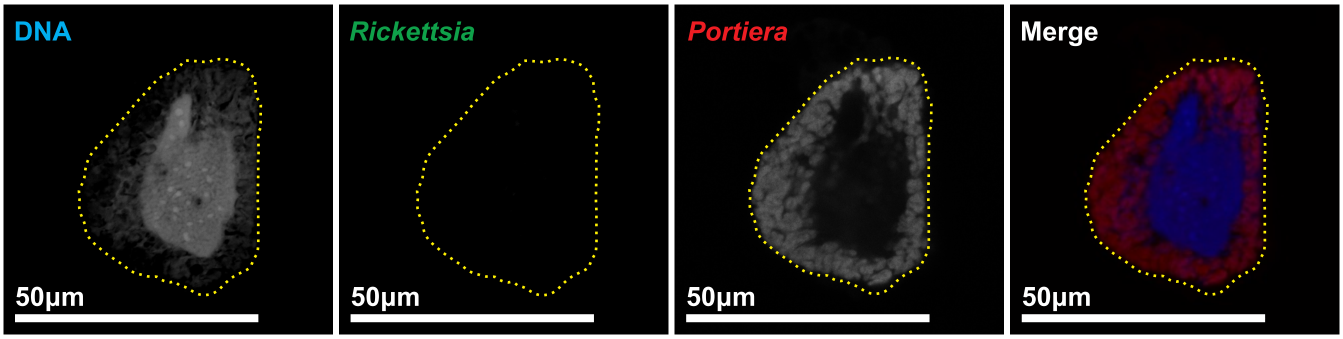

Fig. S2. Confirmation of the absence of *Rickettsia* in bacteriocytes dissected from an adult MED Q2 female (1 d after eclosion). Yellow dashed lines indicate outlines of the bacteriocytes.

Supplementary Fig. S3

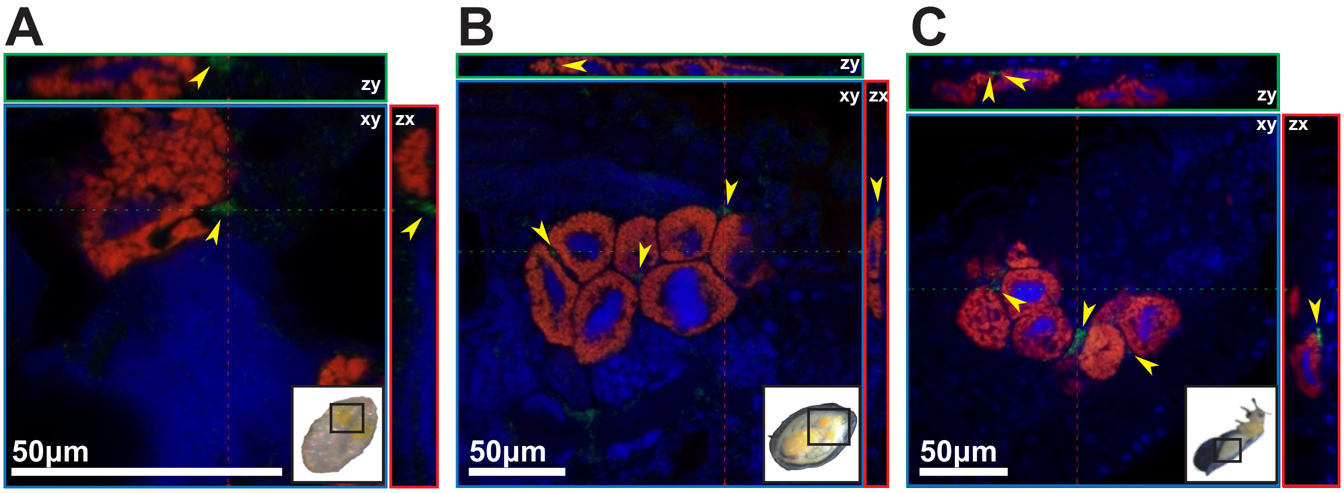

Fig. S3. *In vivo* localisation of *Rickettsia* (green) around bacteriocytes in *Bemisia tabaci* MED Q2. (A) The first-instar and (B) fourth-instar nymph. (C) Male 1 d after eclosion. In each panel, the overall view of *B.tabaci* is arranged on the lower right. The areas marked in black lines were observed using fluorescence in situ hybridization. Orthogonal views of Z-stack images are shown. Red and green dashed lines indicate corresponding points in the orthogonal planes. *Portiera* is shown in red. Host nuclear DNA is visualised in blue. The Yellow arrowhead indicates *Rickettsia* aggregated in close proximity to bacteriocytes.

Supplementary Table S1. *Bemisia tabaci* samples used in this study

Locality no.	Sample locality (City, Prefecture)	Year	Host plant	N (%)			
				Total	MED Q1	MED Q2	JpL
1	Omitama, Ibaraki	2016	cherry tomato	4	4 (100.0)	0 (0.0)	0 (0.0)
2	Tsukuba, Ibaraki	2016	eggplant, tomato	20	12 (60.0)	8 (40.0)	0 (0.0)
3	Sakura, Tochigi	2017	eggplant	2	2 (100.0)	0 (0.0)	0 (0.0)
4	Shioya, Tochigi	2018	tomato	20	20 (100.0)	0 (0.0)	0 (0.0)
5	Utsunomiya, Tochigi	2017	cucumber, tomato	10	6 (60.0)	4 (40.0)	0 (0.0)
6	Kanuma, Tochigi	2017	eggplant	7	0 (0.0)	7 (100.0)	0 (0.0)
7	Mooka, Tochigi	2017	eggplant	18	2 (11.1)	16 (88.8)	0 (0.0)
8	Shimotsuke, Tochigi	2017	cucumber	5	1 (20.0)	4 (80.0)	0 (0.0)
9	Oyama, Tochigi	2018	cucumber	7	1 (14.3)	6 (85.7)	0 (0.0)
10	Kazo, Saitama	2017	cucumber, tomato	10	6 (60.0)	4 (40.0)	0 (0.0)
11	Isesaki, Gunma	2018	cucumber	87	86 (98.9)	1 (1.1) †	0 (0.0)
		2019	eggplant	11	10 (90.9)	1 (9.1) †	0 (0.0)
		2020	cucumber	40	36 (90.0)	4 (10.0) †	0 (0.0)
			eggplant	100	93 (93.0)	7 (7.0) †	0 (0.0)
			spider flower	12	12 (100.0)	0 (0.0)	0 (0.0)
12	Fujioka, Gunma	2017	tomato	4	4 (100.0)	0 (0.0)	0 (0.0)
13	Takasaki, Gunma	2020	squash	2	2 (100.0)	0 (0.0)	0 (0.0)
14	Maebashi, Gunma	2018	squash	3	3 (100.0)	0 (0.0)	0 (0.0)
			tomato	26	26 (100.0)	0 (0.0)	0 (0.0)
		2019	potato	3	1 (33.3)	2 (66.7) *	0 (0.0)
			tomato	25	19 (76.0)	6 (24.0) †	0 (0.0)
		2020	bell pepper	16	16 (100.0)	0 (0.0)	0 (0.0)
			eggplant	13	13 (100.0)	0 (0.0)	0 (0.0)
			potato	5	4 (80.0)	1 (20.0) †	0 (0.0)
			squash	22	19 (86.4)	3 (13.6)	0 (0.0)
			tomato	19	19 (100.0)	0 (0.0)	0 (0.0)
		2021	eggplant	18	13 (72.2)	2 (11.1) †	3 (16.7)
			sweet potato	28	10 (35.7)	4 (14.3) †	14 (50.0)
			tomato	8	1 (12.5)	1 (12.5) †	6 (75.0)
			yacon	15	0 (0.0)	0 (0.0)	15 (100.0)
15	Yoshioka, Gunma	2020	tomato	8	0 (0.0)	0 (0.0)	8 (100.0)
		2021	tomato	6	3 (50.0)	1 (16.7) †	2 (33.3)

* Laboratory strain was established from the sample. The partial 16S rRNA sequences of the symbionts were determined from the strain.

† The partial *mtCOI* sequences were determined from the strains.

Supplementary Table S2. Primers and probes used in this study.

Target organism	Target gene	Primer's name	Sequence (5'→3')	Annealing temperature (°C)	Products size (bp) / Fluorechrome	References[a]
Mix for genotyping						
B.tabaci MEAM1	*mtCOI*	BCOI-324F	TTCTTCTGTAGATGTGTGTC	60	259	1
		BQOI-582R	GCAATCAGCATAATCTGAATATCGA			1
B.tabaci MED Q1	*mtCOI*	Q1COI-158F	CTTCAGCTACTATGATTATTGCC		425	1
B.tabaci MED Q2	*mtCOI*	Q2COI-431F	GATTTCCATTAATCTTGGGT		152	1
B.tabaci MED Q1and Q2	*mtCOI*	QCOI-582R	ACAATCAGCATAATCTGAATATCGG			1
B.tabaci AsiaII 6	*mtCOI*	AsiaII6COI-146F	GGGCTTATTTTACTTCAGCTAC		108	1
		AsiaII6COI-253R	GCGGACTAAACCTGTTAGAT			1
B.tabaci (Universal)	ITS1	TW81	GTTTCCGTAGGTGAACCTGC		541	2
		B.tabaci 5.8R	ATCCGCGAGCCGAGTGATCC			3
Mix1 for symbionts						
Hamiltonella	*16s rRNA*	*Hamiltonella* 16S rDNA-f	GCATCGAGTGAGCACAGTTT	60	293	4
Wolbachia	*16s rRNA*	Wol16S-f	CGGGGGAAAAATTTATTGCT		153	5
Universal	*16s rRNA*	EUB338	GCTGCCTCCCGTAGGAGT			6
Arsenophonus	*16s rRNA*	Ars-435F	AGTCGTGAGGAAGGTGTTAG		419	1
		Ars-853R	AGGCCACAGTCCATGACCA			1
Portiera	*16s rRNA*	Por-366F	GGGAACCCTGATCCAGT		772	1
		1098R	AAAGTTCCCGCCTTATGCGT			7
Mix2 for symbionts						
Rickettsia	*16s rRNA*	Ric-28F	ATGCAAGTCGAACGGACTAAT	60	283	1
Hemipteriphilus	*16s rRNA*	Hemi-140F	CTAATACCGTATGTCCTCGGGA		173	1
Universal	*16s rRNA*	EUB338	GCTGCCTCCCGTAGGAGT			6
Cardinium	*16s rRNA*	Car-457F	GGGGTTCTTGAGAGTACTGTAA		405	1
		Car-861R	GATCACTTAACGCTTTCGCTT			1
Portiera	*16s rRNA*	Por-366F	GGGAACCCTGATCCAGT		772	1
		1098R	AAAGTTCCCGCCTTATGCGT			7
Primers for sequencing						
B.tabaci	*mtCOI*	C1-J-2195	TTGATTTTTTGGTCATCCAGAAGT	45	802	8
		TL2-N-3014	TCCAATGCACTAATCTGCCATATTA			8
Eubacteria	*16s rRNA*	16SA1	AGAGTTTGATCMTGGCTCAG	55	approx. 1,500	9
		16SB1	TACGGYTACCTTGTTACGACTT			9
Primers for qPCR						
Portiera	*16s rRNA*	BTP1	TGTCAGTGTCAGCCCAGAAG	55	157	10
		Portiera 16S rDNA-R-comp	TTTATGTGAAAGCCCTATGCTTA			11
Rickettsia	*gltA*	*Rickettsia gltA* -F	AAAGGTTGCTCATCATGCGTT	60	80	12
		Rickettsia gltA -R	GCCATAGGATGCGAAGAGCT			12
TYLCV	*v1*	Qv1F447	CAGCCCAATGGATTTTGGAC	65	216	13
		Qv1R662	TACTTGGCTGCCTCCTGATG			13
Mix for TYLCV linage discrimination						
TYLCV Israel (IL) and mild (Mld)	*C1*	TYLCV-1840F	GGTCTACGTCATCAATGAC	55	–	14
TYLCV IL	–	IL-2642R	ACACCGATTCATTTCAAC		802	14
TYLCV Mld	*C4*	Mld-2354R	AGGGAGCTAAATCCAGTT		514	14
FISH Probes						
Portiera	*16s rRNA*	Alexa555-BTP1	TGTCAGTGTCAGCCCAGAAG		Alexa Fluor 555	10
Rickettsia	*16s rRNA*	Alexa488-Rb1	TCCACGTCGCCGTCTTGC		Alexa Fluor 488	10

[a] 1.Kurata et al., *Appl. Entomol. Zool*. 51, 167-172 (2016); 2.Joyce et al., COST 812 Biotechnology: Genetics of entomopathogenic nematode-bacterium complexes. Burnell AM, Ehlers RU, Masson JP, (eds.). Proc. Symp. Workshop, St. Patrick's College, Maynooth, Co. Kildare, Ireland. European Commission, DG XII, Luxembourg, 178–187 (1994); 3.De Barro et al., *Mol. Phylogenet. Evol.* 16, 29-36 (2000); 4.Brumin et al., *Insect Sci.* 18, 57-66 (2011); 5.Heddi et al., *Proc. Natl. Acad. Sci. USA.* 96, 6814–6819 (1999); 6.Amann et al., *Appl. Environ. Microbiol.* 56, 1919–1925 (1990); 7.Zchori-Fein et al., *Ann. Entomol. Soc. Am.* 95, 711-718 (2002); 8.Simon et al., *Ann. Entomol. Soc. Am.* 87, 651–701 (1994); 9.Fukatsu & Nikoh, *Appl. Environ. Microbiol.* 64, 3599-3606 (1998); 10.Gottlieb et al., *Appl. Environ. Microbiol.* 72, 3646-3652 (2006); 11.Fujiwara et al., *Microbiol. Spectr.* 11, e0468422; 10.1128/spectrum.04684-22 (2023); 12.Ghanim et al., *Pest Manag. Sci.* 65, 939-942 (2009); 13.Ohnishi et al., *J. Gen. Plant Pathol.* 75, 131-139 (2009); 14.Lefeuvre et al., *J. Virol. Methods.* 144, 165-168 (2007).
